# Functionalisation of PLLA nanofiber scaffolds using a possible cooperative effect between collagen type I and BMP-2: impact on colonization and bone formation in vivo

**DOI:** 10.1007/s10856-012-4697-0

**Published:** 2012-06-21

**Authors:** Markus D. Schofer, Lisa Tünnermann, Hendric Kaiser, Philip P. Roessler, Christina Theisen, Johannes T. Heverhagen, Jacqueline Hering, Maximilian Voelker, Seema Agarwal, Turgay Efe, Susanne Fuchs-Winkelmann, Jürgen R. J. Paletta

**Affiliations:** 1 Department of Orthopedics and Rheumatology, University Hospital Marburg, Baldingerstraße, 35043 Marburg, Germany; 2 Department of Radiology, University Hospital Marburg, Baldingerstraße, 35033 Marburg, Germany; 3 Department of Macromolecular Chemistry, Philipps-University Marburg, Hans-Meerwein-Straße, 35032 Marburg, Germany; 4 Department of Orthopedics and Rheumatology, Philipps-University, Baldingerstraße, 35043 Marburg, Germany

## Abstract

The reconstruction of large bone defects after injury or tumor resection often requires the use of bone substitution. Artificial scaffolds based on synthetic biomaterials can overcome disadvantages of autologous bone grafts, like limited availability and donor side morbidity. Among them, scaffolds based on nanofibers offer great advantages. They mimic the extracellular matrix, can be used as a carrier for growth factors and allow the differentiation of human mesenchymal stem cells. Differentiation is triggered by a series of signaling processes, including integrin and bone morphogenetic protein (BMP), which act in a cooperative manner. The aim of this study was to analyze whether these processes can be remodeled in artificial poly-(l)-lactide acid (PLLA) based nanofiber scaffolds in vivo. Electrospun matrices composed of PLLA-collagen type I or BMP-2 incorporated PLLA-collagen type I were implanted in calvarial critical size defects in rats. Cranial CT-scans were taken 4, 8 and 12 weeks after implantation. Specimens obtained after euthanasia were processed for histology and immunostainings on osteocalcin, BMP-2 and Smad5. After implantation the scaffolds were inhomogeneously colonized and cells were only present in wrinkle- or channel-like structures. Ossification was detected only in focal areas of the scaffold. This was independent of whether BMP-2 was incorporated in the scaffold. However, cells that migrated into the scaffold showed an increased ratio of osteocalcin and Smad5 positive cells compared to empty defects. Furthermore, in case of BMP-2 incorporated PLLA-collagen type I scaffolds, 4 weeks after implantation approximately 40 % of the cells stained positive for BMP-2 indicating an autocrine process of the ingrown cells. These findings indicate that a cooperative effect between BMP-2 and collagen type I can be transferred to PLLA nanofibers and furthermore, that this effect is active in vivo. However, this had no effect on bone formation. The reason for this seems to be an unbalanced colonization of the scaffolds with cells, due to insufficient pore size.

## Introduction

Surgical reconstruction of bone defects frequently requires the use of graft material. Beside autologous bone grafts, artificial bone grafts based on synthetic biomaterials such as metals, polymers, porous ceramics, hydroxyapatite, collagen sponges or hydrogels, as well as several composites, have been developed recently [1–3]. Among them scaffolds based on nanofibers offer several advantages [4–6]. They are thought to mimic the extracellular matrix (ECM) [7–10] and allow differentiation of human mesenchymal stem cells (hMSC) towards osteoblasts. However, this process depends on the chemical composition of the nanofibers. In comparison to other α-(hydroxyester)-based nanofibrous scaffolds, poly-(l)-lactide acid (PLLA) scaffolds support the highest rate of proliferation of mesenchymal stem cells [11] with a tendency to higher cell densities [12, 13]. An initial down regulation of genes associated with the osteoblast linage [12, 14] can be eliminated by functionalisation of the nanofibers either by blending with collagen type I [15] or incorporation of recombinant bone morphogenic protein 2 (BMP-2) in vitro [12]. With respect to bone formation, PLLA nanofiber scaffolds are superior compared to solid wall scaffolds in vivo, due to the nanofibrous geometry [16]. Addition of BMP-2 enhances bone formation, independent of whether BMP is incorporated directly [17], in core–shell nanotubes [18] or loaded in PLGA/HAp nanofibers [19].

Bone regeneration is controlled by a variety of growth factors [20, 21], as well as interactions with the ECM. It is known that the adhesion to collagen I via α2β1 integrin is sufficient to induce osteogenic differentiation of hMSC′s, even in the absence of exogenous stimuli [22, 23]. Furthermore evidence can be found that this integrin signaling pathway is influenced by the BMP-2/Smad signaling. BMP-2 induces formation of integrins on osteoblasts [24, 25]. This, in interaction with the ECM, influence the BMP-function [25] resulting in migration, activation of focal adherence kinase and formation of focal adhesion [26, 27]. Suzawa et al. [28] found evidence, that the downstream pathway of integrin is involved in Smad1 signals, activated by BMP-2 and provides a possible mechanism for cooperation between intracellular signals activated by integrin and BMP receptors in osteoblastic cells. We recently transferred this cooperative effect between collagen and BMP signaling pathway into PLLA based nanofiber scaffolds. In vitro, these scaffolds exhibit a strong osteoinductive potential, accompanied by an up regulation of focal adherence kinase and Smad5, key proteins in BMP-2 and integrin signaling [29].

In order to analyze whether this cooperative effect can be induced by BMP-2 enwoven PLLA-collagen type-I nanofibers in vivo, scaffolds were implanted into critical size defects in rats and analyzed with respect to bone formation in a time dependent manner.

## Materials and methods

### Construction of nanofibers and characterization

The preparation of PLLA-collagen type I blend nanofibers, as well as the incorporation of BMP-2 into PLLA nanofibers by electrospinning, has been reported earlier [29]. Briefly, PLLA and collagen were dissolved in hexafluoroisopropanol (HFIP) in a 4:1 ratio resulting in a 4.5 % (w/v) polymer solution. The spinning solution had a surface tension of 18.33 ± 0.03 mN/m, a viscosity of 1.529 PaS and a conductivity of ~4.7 μs/cm. Spinning process was performed at a flow rate of 14 μl/min with an applied voltage of 10–18 kV and an electrode distance of 15 cm. To incorporate BMP-2 into the nanofibers, 25 μg lyophilized BMP-2 (Reliatech, Braunschweig Germany) was dissolved in 125 μl 50 mM acetic acid and stabilized by addition of 25 μl fetal calf serum (FCS). This mixture was emulsified in 2.5 ml of a 4.5 % PLLA-collagen type I solution (4:1) over a period of 1 min using a vortex mixer (MS 2 Minishaker IKA^®^; 2500 rpm). The spinning process was performed at a flow rate of 14 μl/min with an applied voltage of 10–18 kV and a distance of 15 cm.

### Animals

Sixty, five-month-old Sprague–Dawley rats (Harlan Winkelmann, Borchen, Germany) were used in the experiment. The animals were kept in individual plastic cages (Macrolon Type III) in a room held at a constant temperature of 22.1 °C, with a 12 h light/dark cycle. They had free access to water and standard laboratory pellets (LASQCdiet® Rod16 Rad, LASVendi, Soest, Germany). All experiments were carried out in accordance with the recommendations in the Guide for the Care and Use of Laboratory Animals of the NIH and approved by the local Animal Ethics Committee (V 54–19 c 20–15 (1) MR 20/21- Nr. 34/2009).

Following the 3-R principle (replace, reduce and refine) for animal testing a former control group (empty defect) was not re-operated. All animals were operated by the same surgeon within 12 month and healing outcomes were followed over a period of 12 weeks.

### Surgery

Animals were divided in two groups and surgeries were preformed as described earlier [17]. Defects were filled with either PLLA-collagen type-I or BMP-2 enwoven PLLA-collagen type-I nanofiber scaffolds.

### Dual-source CCT

Radiographic evaluation was done under general anesthesia 4, 8 and 12 weeks after surgery using cranial computer tomography (CCT) imaging (Somatom Definition, Siemens Medical Systems, Erlangen, Germany) with a resolution of 0.3 mm. Radiological density was measured by placing a region of interest (ROI) of the same size as the defect over each data set (Leonardo, Siemens Medical Systems, Erlangen, Germany).

### Harvesting of tissue and sectioning of test specimens

Animals of each group were sacrificed by CO_2_-asphyxiation after 4, 8 and 12 weeks. Blood samples were taken from animals sacrificed after 4 weeks and inflammatory parameters like C-reactive protein (CRP) and haptoglobin were analyzed. The defect sites were removed from dead animals together with a small amount of surrounding bone, skin and soft connective tissue. These samples were immediately fixed in 4 % buffered formalin for 3 days and then decalcified in an EDTA-solution (Osteosoft^®^, Merck, Darmstadt, Germany) over a period of 18 days. After trimming the bone specimens with a precision saw, they were dehydrated in graded alcohol solution and cedar wood oil and embedded in paraffin. Sections were cut at 5 μm with a 40° stainless-steel blade on a rotation microtome (RM2055, Leica Microsystems, 158 Bensheim, Germany).

### Histological and immunohistological staining

After removal of paraffin using Xylene and re-hydration by decreasing alcohol series histological staining was performed with Masson Goldner (MG) formulations according to standard protocols. For immunohistological staining endogenous peroxidase activity was quenched with a 4 % hydrogen peroxide solution after re-hydration. Normal horse serum (Santa Cruz, Heidelberg, Germany) was used in order to block unspecific bindings. Then slices were incubated over night with a polyclonal IgG antibody against either osteocalcin (1:50; FL-100, Santa Cruz, Heidelberg, Germany), BMP-2 (1:25; N-14, Santa Cruz Heidelberg, Germany) or Smad5 (1:25; D-20, Santa Cruz Heidelberg, Germany). Sections were then incubated with a biotinylated secondary antibody (Santa Cruz, Heidelberg, Germany) diluted 1:50 for 30 min at room temperature. An avidin–biotin complex detection system coupled with DAB as a chromogen (Santa Cruz, Heidelberg, Germany) was used to visualize antibody binding after 20 min incubation time at room temperature. Finally, all sections were counterstained with Gill’s hematoxylin solution (Santa Cruz, Heidelberg, Germany) diluted 1:2 for 10 s. Negative controls without primary antibody incubation were carried along together with each of the previously described staining.

### Histological analysis

Histological analysis was carried out using a digital microscope (DM5000, Leica Microsystems, Bensheim, Germany) and QUIPS analysis software (Leica Microsystems, Bensheim, Germany) as described in [17]. Cell counts were performed in four fields of at least six specimens, ranging from one defect end to the other, using primary magnifications of 40/20 fold.

### Statistical analysis

Analysis of variance (ANOVA) was used to evaluate the differences between experimental groups and control group as well as between different points of time in each group. Data are given as means ± standard deviations. The level of significance was set at *P* < 0.05.

## Results

Four animals had to be sacrificed 1 day after surgery due to neurological deficits but all remaining animals survived and the implant sites healed well. Body weight increased during 3 month from 278 to 435 g in the PLLA-collagen group and from 279 to 441 g in the PLLA-collagen-BMP group. Serum blood analysis of CRP as well as haptoglobin yielded no signs of infection or inflammation. Levels of 9.67 ± 0.98 and 9.84 ± 1.99 mg/L of CRP and 0.97 ± 0.30 and 0.78 ± 0.33 mg/dL of haptoglobin were found in the PLLA-collagen group and in the PLLA-collagen-BMP-2 group 4 weeks after surgery, without significant differences between the groups. Animals receiving either PLLA-collagen or PLLA-collagen-BMP-2 nanofiber scaffolds showed firm fixation of the implants on palpation.

### Effects on colonization

**Fig. 1:**
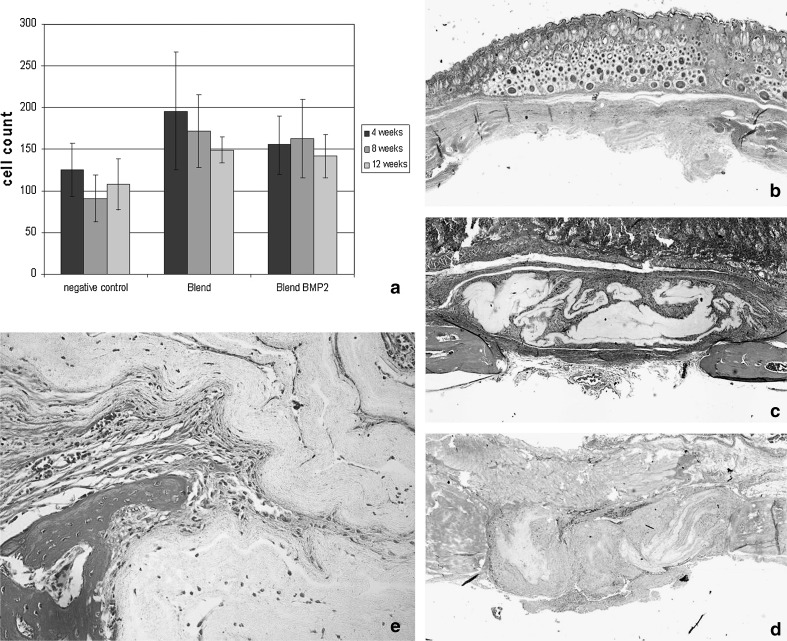
Influence of nanofiber implant on cell densities and callus formation Mean cell densities (**a**), and Microphotographs of masson goldner stained samples of negative control (**b**), PLLA-collagen type I (**c**), and PLLA-collagen type I/BMP-2 (**d**), 12 weeks after implantation into critical size defects. Detail screen showing cells accumulated mainly in wrinkles or channels (**e**)

**Fig. 2:**
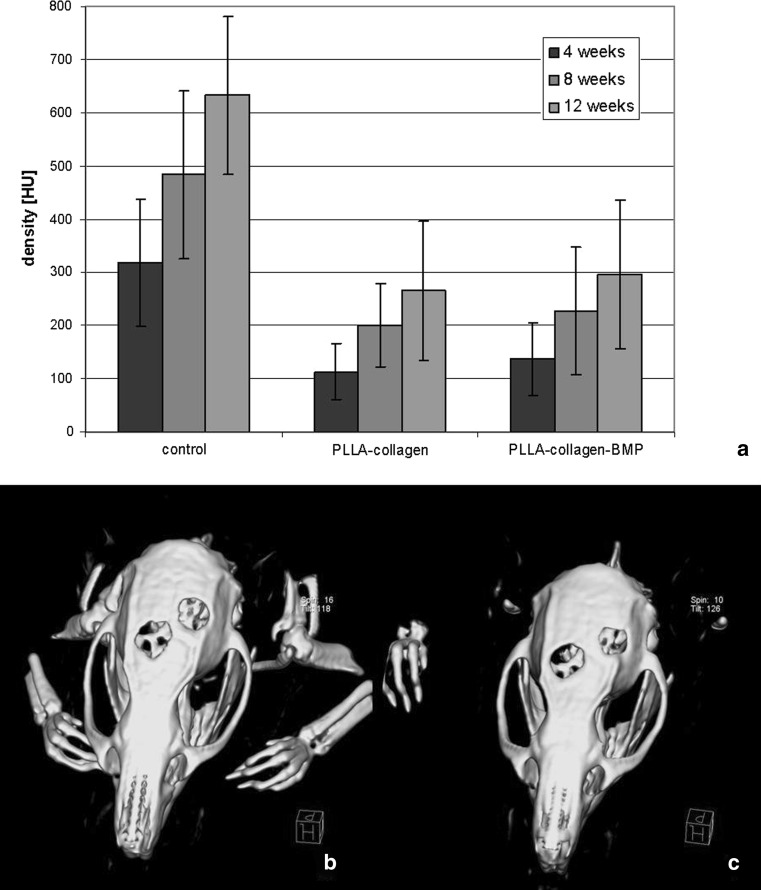
Influence of nanofiber implant on defect calcification Radiological density [HU] of defect areas measured by cranial CT-scans (**a**), and 3D reconstructions of cranial CT-scans (**b**), PLLA-collagen type I (**c**), and PLLA-collagen type I/BMP-2, each 4 weeks after implantation

To analyze the impact of PLLA-collagen type-I and BMP-2 enwoven PLLA-collagen type-I nanofibers on bone healing, we performed total cell count, analyzed the formation of hard callus determined by Masson Goldner staining and determined bone formation using CT scans. As shown in Fig. 1a only a few cells migrate into the scaffold resulting in a slight increase as compared to control defect. It is notable that the distribution of the cells was inhomogenous. Cells were accumulated within wrinkles or channels throughout the scaffold leaving the main part un-colonized (Fig. 1e). Furthermore formation of hard callus failed to appear in most areas of the defect as determined by Masson Goldner staining (Fig. 1c, d and Fig. 1b control). These findings were supported by CT-scans. Within three month, bone density was lower as compared to empty controls with no significant differences between the two treatment groups (Fig. 2).

**Fig. 3:**
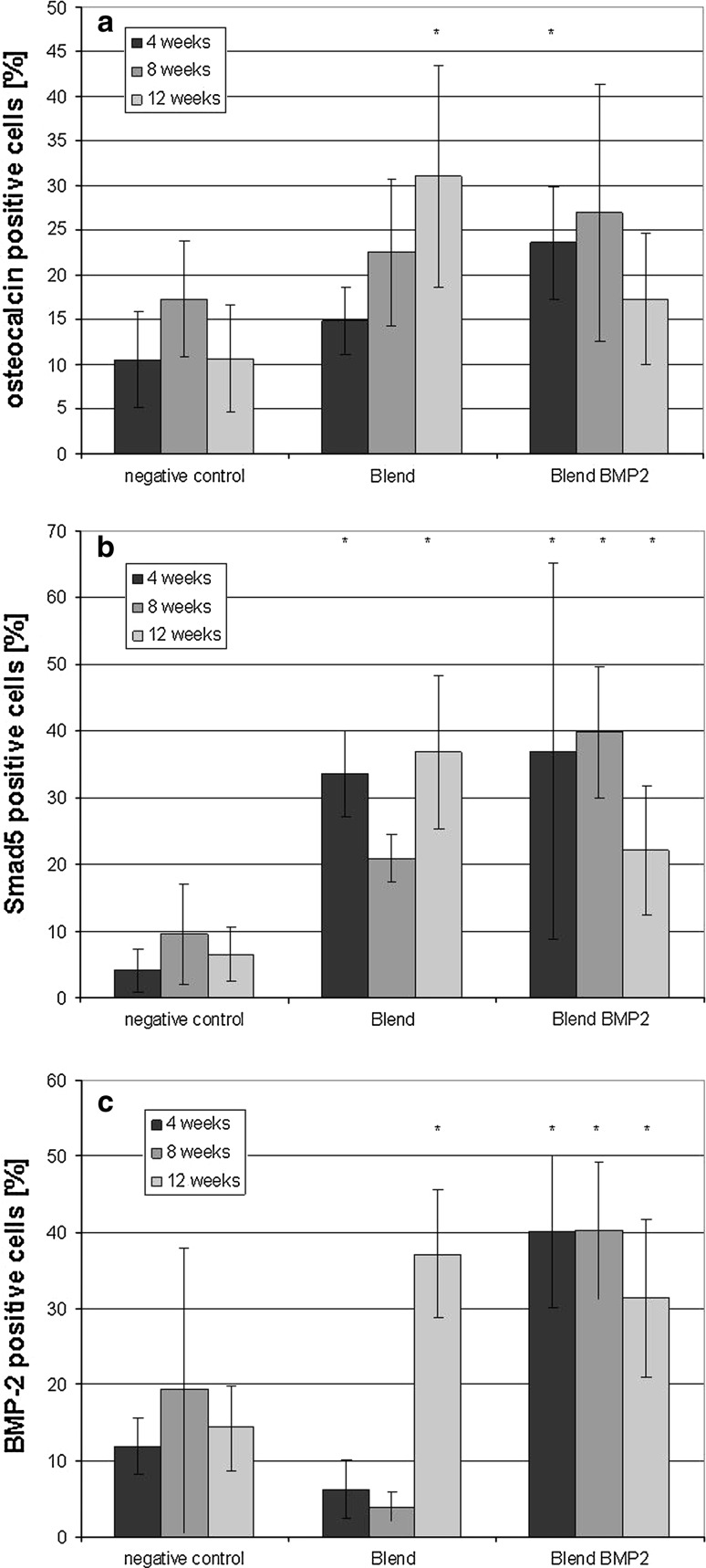
Time dependent influence of nanofiber implant on osteocalcin (**a**) Smad5, (**b**) and BMP-2, (**c**) positive cells as determined by immunhistochemistry

To analyze whether this delay in bone formation is either due to a missing cell migration or down regulation of osteocalcin, we next determined the amount of osteocalcin positive cells within the scaffold. As shown in Fig. 3a more osteocalcin positive cells were detected when either PLLA-collagen type-I or BMP-2 enwoved PLLA-collagen type-I nanofibers were implanted into the defect as compared to empty defects. However significant differences were detected after 4 weeks in the BMP-2 enwoven PLLA-collagen group and after 12 weeks in the PLLA-collagen group.

The maintenance of the osteoblast phenotype was analyzed by determining the amount of Smad5 and BMP-2 positive cells within each scaffold. As shown in Fig. 3b three times more Smad5 positive cells were found in PLLA-collagen nanofiber scaffold compared to empty defects. Here the incorporation of BMP-2 had no further impact on Smad5 expression. Nevertheless, focusing on BMP-2 positive cells the PLLA-collagen nanofiber scaffolds had only late stage effects. Here incorporation of BMP-2 results in significant more BMP-2 expressing cells, especially in early stages of defect healing (Fig. 3c).

## Discussion

Tissue-engineered scaffolds should be analogous to native ECM in terms of both chemical composition and physical structure. Polymeric nanofiber matrix, with its nanoscaled nonwoven fibrous structure is a suitable material to mimic ECM [30]. Nevertheless, the polymer itself influences cell behavior. PLLA nanofibers allow cell growth and differentiation although an initial down regulation of genes associated with the osteoblast lineage can be observed [12, 14]. It had been shown earlier that this effect can be overcome by functionalisation of nanofibers either by BMP incorporation or blending with collagen type I in vitro [12, 15]. PLLA nanofiber scaffolds––implanted into critical size defects––were colonized homogeneously by cells and induced bone formation when functionalized with BMP-2 [17]. In contrast, the PLLA-collagen nanofiber scaffolds we implanted into critical size defects were less colonized and distribution of cells was inhomogeneous and restricted to wrinkles or channels. This was accompanied by reduced bone densities and hard callus formation––independent of BMP-2 incorporation.

However, the increased ratio of osteocalcin positive cells––localized within the scaffolds––shows that the osteoinductive potential observed in vitro [15, 29] is maintained in vivo in principal. Furthermore, the use of PLLA-collagen type I nanofiber scaffolds resulted in an enhanced staining of Smad5 as compared to controls. In addition approximately 40 % of the cells stained positive for BMP-2 when BMP-2 enwoven PLLA-collagen type-I scaffolds were implanted. This was more as compared to the corresponding scaffolds without BMP-2, as well as to PLLA and BMP-2 enwoven PLLA nanofiber scaffolds as published earlier [17]. These findings indicate that the cooperative effect between BMP-2 and collagen type-I as postulated by [28] can be transferred to PLLA nanofibers and theoretically maintains in vivo.

Despite the osteoinductive potential, the scaffolds did not induce bone formation in vivo due to poor cell colonization which can be interpreted as a tribute to the pore size of the scaffolds. As reported earlier PLLA-collagen type-I nanofiber scaffolds exhibited a pore size of 2 μm which is reduced after BMP-2 incorporation [29]. To achieve bone ingrowths into porous scaffolds pore sizes of approximately 50–125 μm [31] or even higher [32] had been reported. In case of nanofiber scaffolds pore sizes seem to play another role. A recently published review [33] comparing different nanofiber/cell combinations found optimal pore diameter for nanofiber scaffolds ranging from 5 to 50 μm. With respect to PLLA nanofibers produced from DCM pore diameter of 30 μm were reported [34] while PLLA-collagen blend nanofiber scaffolds showed pore diameter below 2 μm [35]. Here the low pore diameter might be one reason for the lack of colonization.

Another reason might be the presence of solvent residues. In gelatin blended PCL nanofibers HFIP residues up to 16,600 ppm were detected, depending on the gelatin content and treatment of the fibers [36] while DCM residues in PLGA nanofibers were about 400 ppm [19]. Bearing in mind that in vitro cells proliferate and differentiate on PLLA nanofiber scaffolds using both HFIP or DCM as solvent, solvent residues seem to play a minor part in vivo.

Consequently, the absence of bone formation is not due to inappropriate functionalisation of the PLLA-collagen scaffolds, but more likely a result of unbalanced colonization of the scaffolds with cells due to insufficient pore size.

If it is possible to overcome limitations of pore sizes, either by sacrificial fibers, [37, 38], variations in fiber diameter [39], incorporation of salt or ice crystals [36, 40] or direct incorporation of cells [41] the scaffolds presented here could serve as a suitable implant for bone repair.
